# Ripasudil as a Potential Therapeutic Agent in Treating Secondary Glaucoma in HTLV-1-Uveitis: An In Vitro Analysis

**DOI:** 10.3390/ijms25063229

**Published:** 2024-03-12

**Authors:** Mingming Yang, Koju Kamoi, Yuan Zong, Jing Zhang, Yaru Zou, Kyoko Ohno-Matsui

**Affiliations:** Department of Ophthalmology & Visual Science, Graduate School of Medical and Dental Sciences, Tokyo Medical and Dental University, Tokyo 113-8510, Japan; yangmm12.oph@tmd.ac.jp (M.Y.); zongyuan666.oph@tmd.ac.jp (Y.Z.); zhangjing.oph@tmd.ac.jp (J.Z.); alicezouyaru519@gmail.com (Y.Z.); k.ohno.oph@tmd.ac.jp (K.O.-M.)

**Keywords:** human T-cell leukemia virus type 1, HTLV-1 uveitis, glaucoma, ocular inflammation, ripasudil, Rho-associated coiled coil-forming protein kinase inhibitor

## Abstract

Human T-cell leukemia virus type 1 (HTLV-1), a virus that affects 5–10 million people globally, causes several diseases, including adult T-cell leukemia-lymphoma and HTLV-1-associated uveitis (HU). HU is prevalent in Japan and often leads to secondary glaucoma, which is a serious complication. We investigated the efficacy of ripasudil, a Rho-associated coiled coil-forming protein kinase inhibitor, in alleviating changes in human trabecular meshwork cells (hTM cells) infected with HTLV-1. HTLV-1-infected hTM cells were modeled in vitro using MT-2 cells, followed by treatment with varying concentrations of ripasudil. We assessed changes in cell morphology, viability, and inflammatory cytokine levels, as well as NF-κB activation. The results showed that ripasudil treatment changed the cell morphology, reduced the distribution of F-actin and fibronectin, and decreased the levels of certain inflammatory cytokines, such as interleukin (IL)-6, IL-8, and IL-12. However, ripasudil did not significantly affect NF-κB activation or overall cell viability. These findings suggest that ripasudil has the potential to treat secondary glaucoma in patients with HU by modulating cytoskeletal organization and alleviating inflammation in HTLV-1-infected hTM cells. This study lays the foundation for further clinical studies exploring the effectiveness of ripasudil for the treatment of secondary glaucoma associated with HU.

## 1. Introduction

Human T-cell leukemia virus type 1 (HTLV-1) was discovered in 1980 and has been reported to have infected humans for thousands of years [1]. Currently, HTLV-1 has affected more than 5–10 million people worldwide [2] and can cause many diseases, including adult T-cell leukemia-lymphoma (ATL), HTLV-1-associated myelopathy (HAM), and HTLV-1-associated uveitis (HU) [3,4,5], which is the second most common HTLV-1-associated disease. Japan has the largest number of HTLV-1 infections in the world [3]. According to cross-sectional studies, the prevalence of HU in Japan is estimated to be 112.2/100,000 HTLV-1 carriers [4]. In southern Japan, HU is the most prevalent type of uveitis and is considered one of the most important ocular diseases in endemic areas [5]. Recently, the number of people infected with HTLV-1 has increased due to horizontal transmission in metropolitan areas [3]. HU has garnered attention because the horizontal transmission of HTLV-1 can cause its development [6]. Furthermore, a low proviral load (PVL) of HTLV-1 may cause HU in individuals with Graves’ disease [7,8].

Glaucoma is one of the most prevalent secondary HU diseases [9]. Glaucoma is closely associated with uveitis [10]. HTLV-1-infiltrating cells have been detected in the anterior chamber. Our previous study demonstrated that HTLV-1-infected human trabecular meshwork cells (hTM cells) contribute to the accumulation of inflammatory cytokines and chemokines, which recruit a large number of inflammatory cells to the TM. Simultaneously, HTLV-1 infection of TM cells can disrupt their functions [11]. Altogether, it is reasonable to assume that the prevalence of secondary glaucoma in HU is higher than that in general uveitis (10–23%). Nevertheless, only a few studies have focused on secondary glaucoma of HU [11], and research on the treatment of this blindness-threatening disease is lacking.

Rho-associated coiled coil-forming protein kinase (ROCK), the most studied downstream activator of the small GTPase RhoA, is expressed in TM and ciliary muscle cells [12]. The Rho/ROCK signaling pathway regulates cell morphology, actin stress fibers, cell-ECM interactions, and cell-cell junctions [13,14]. Recent studies have highlighted the role of the ROCK pathway in viral replication (e.g., buffalopox and vaccinia viruses) and inflammation [15]. Inhibiting the ROCK signaling pathway can improve ocular blood flow and axon regeneration and inhibit retinal ganglion cell apoptosis and scarring after glaucoma filtering surgery [16,17,18,19,20]. A ROCK selective inhibitor, also known as ripasudil hydrochloride hydrate (K115), was approved in Japan in 2014 and has been shown to lower intraocular pressure (IOP) by altering the conventional outflow facility. It has been well established that this ROCK inhibitor can effectively reduce IOP both in vitro and in vivo [13,21,22,23]. Several studies have shown that ripasudil can alleviate LPS-induced inflammation in RPE cells by targeting the miR-136-5p/ROCK/NLRP3 pathway [24,25]. Nevertheless, whether ripasudil inhibits hTM cell changes caused by HTLV-1 infection remains unknown.

In this study, we used MT-2 cells as HTLV-1-infected T cells, established secondary glaucoma in the HU in vitro model, and determined the effect of ripasudil on the morphology and function of HTLV-1-infected hTM cells.

## 2. Results

### 2.1. HTLV-1 Provial Loads after Cocultivation with HTLV-1-Infected Cell

**Figure 1:**
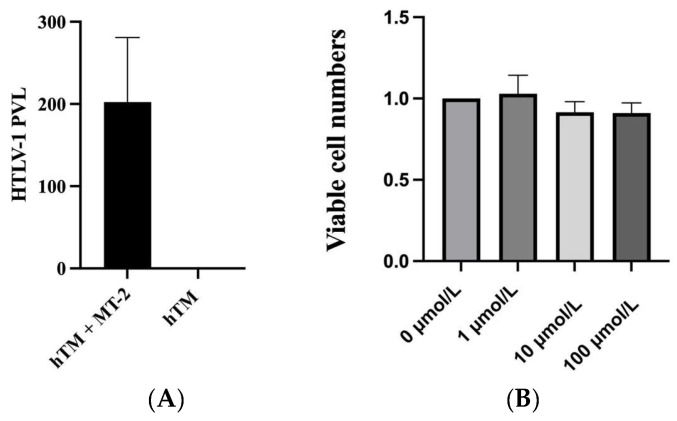
(**A**) Detection of HTLV-1 proviral loads was performed by real-time polymerase chain reaction in the human trabecular meshwork (hTM) cells cultured alone and hTM cells transferred three times after coculturing with MT-2. The hTM cells were placed at a density of 1 × 10^5^ cells in a 12-well plate. The hTM cells were infected three times with MT-2. The data were taken from three independent biological experiments. (**B**) Cell viability was detected by CCK-8 after 60 min treatment with 0, 1, 10, and 100 μmol/L of ripasudil.

We used hTM cells cocultured with irradiated MT-2 cells to build an in vitro secondary glaucoma in the HU model [11,26]. To confirm that hTM cells were infected, we used real-time polymerase chain reaction (PCR) to detect HTLV-1 PVL after cocultivation and three transfers. The results showed that the HTLV-1 PVL of hTM cells significantly increased after cocultivation (Figure 1A). Trypan blue staining on day 6 after irradiation showed that irradiated MT-2 cells were not viable. This result indicated that hTM cells were successfully infected after cocultivation with MT-2 and can be used as an in vitro secondary glaucoma in the HU model.

### 2.2. Effect of Ripasudil on hTM Cell Viability

After ripasudil treatment, the CCK-8 assay was used to evaluate cell viability. Results showed that, compared with hTM cells without treatment, treatment with 1 μmol/L ripasudil hTM cells increased cell viability. However, no statistical difference was found in all three treatment groups compared with the untreated hTM cells (Figure 1B).

### 2.3. Effect of Ripasudil on the Morphology of hTM Cell

**Figure 2:**
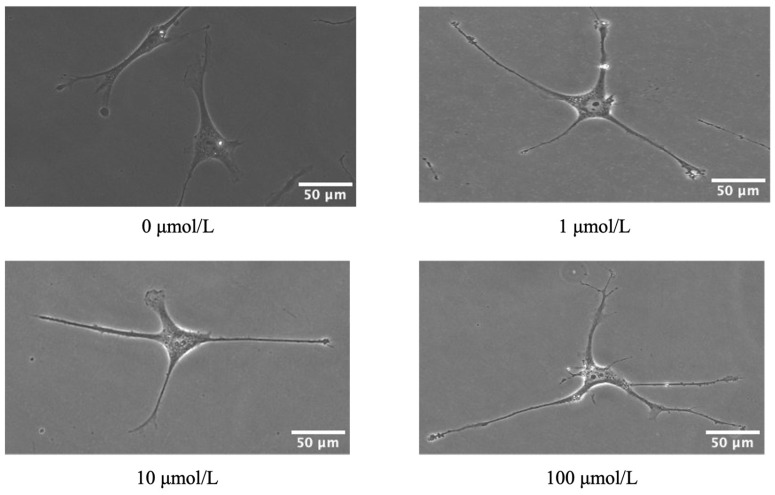
Effect of ripasudil on human trabecular meshwork (hTM) cell morphology. The hTM cells were cultured with 0, 1, 10, and 100, μmol/L ripasudil for 60 min. Ripasudil-induced retraction of hTM cells.

Phase-contrast microscopy was used to directly observe morphological changes in hTM cells. The results showed that hTM cells retracted under the light microscope after 60 min of treatment with ripasudil at concentrations of 1, 10, and 100 μmol/L, compared with untreated hTM cells (Figure 2). We observed that the hTM cells became thinner and smaller after ripasudil treatment.

### 2.4. Effect of Ripasudil on the Cytoskeleton of hTM Cells

**Figure 3:**
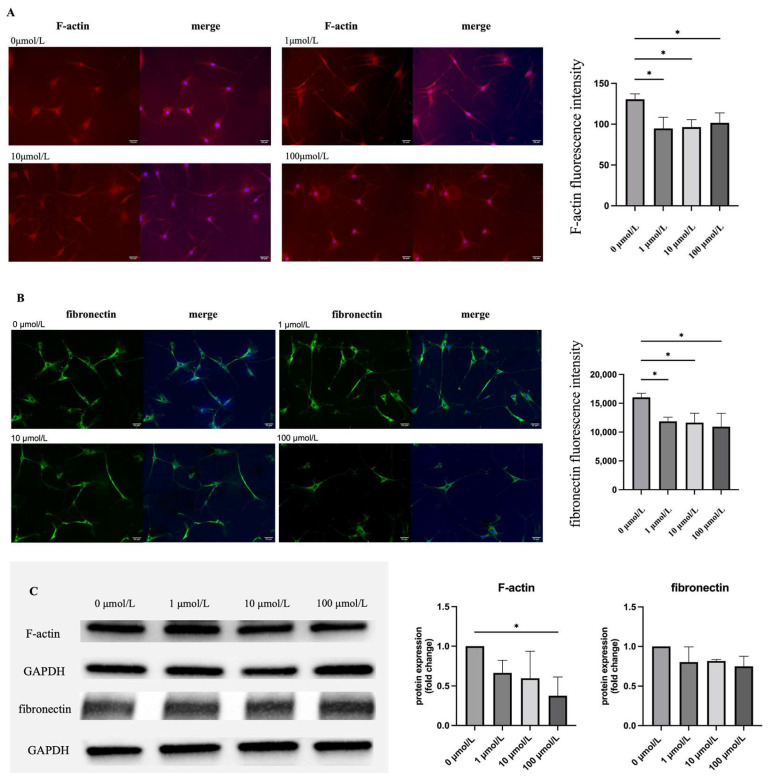
(**A**) Effect of ripasudil on the distribution of F-actin. Infected hTM cells were treated with 0, 1, 10, and 100 μmol/L ripasudil for 60 min. F-actin was stained with green; cell nuclei were counterstained with DAPI (blue). Treatment with ripasudil reduced the distribution of F-actin. Fluorescence intensity was reduced from 130.6 ± 6.6 to 94.8 ± 13.6, 96.45 ± 9.3 and 101.7 ± 12.11, respectively (*p* = 0.0151, *p* = 0.0194, *p* = 0.0442, respectively). (**B**) Effect of ripasudil on the distribution of fibronectin. The distribution of fibronectin (green) and cell nuclei (blue) was shown. Infected hTM cells were treated with 0, 1, 10, 100 μmol/L ripasudil for 60 min. Treatment with ripasudil reduced the distribution of fibronectin. Fluorescence intensity was reduced from 16,032 ± 703.4 to 11,858 ± 730.9, 11,647 ± 1628, and 10,936 ± 2321, respectively (*p* = 0.0381, *p* = 0.0300, *p* = 0.0137, respectively). Scale bar: 50 μm. (**C**) Comparison of F-actin and fibronectin expression in the hTM cell. Representative images are shown on the left. Compared with the hTM cell without treatment, treatment with 100 μmol/L ripasudil significantly reduced the expression of F-actin (*p* = 0.0361) (*: *p* < 0.1).

To investigate cytoskeletal changes in hTM cells after ripasudil treatment, we evaluated the distribution of F-actin and fibronectin. Immunofluorescence and western blot (WB) were used to evaluate the distribution of F-actin (red) and fibronectin (green), and ImageJ software (version 1.54) was used to analyze the fluorescence intensity and WB bands of F-actin and fibronectin. The results showed that, compared to hTM cells without treatment, cells with 1, 10, and 100 μmol/L of ripasudil reduced the distribution of both F-actin and fibronectin. The detailed results are presented in Figure 3.

### 2.5. Effect of Ripasudil on Inflammation Cytokines and Chemokines

**Figure 4:**
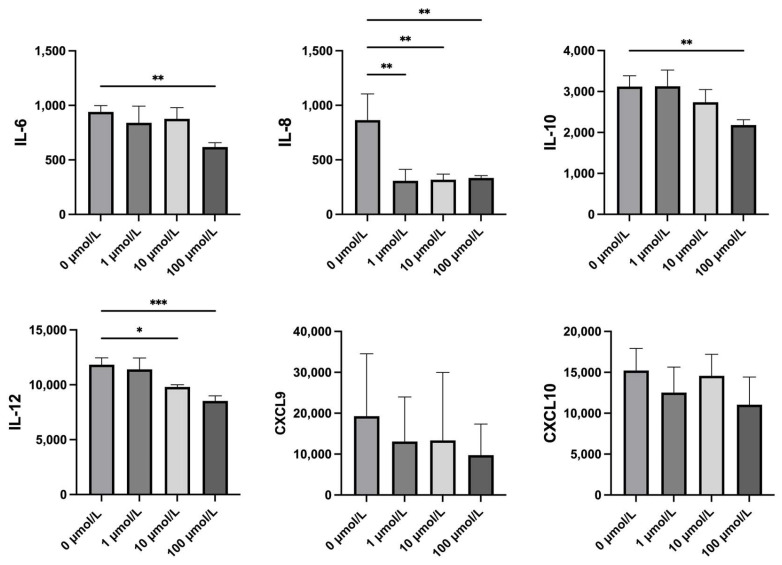
Effect of ripasudil on inflammation cytokines and chemokines. A cytometric bead array was used to detect the level of inflammation cytokines and chemokines in the supernatant of hTM cells cocultured with MT-2. Interleukin(IL)-8 decreased after treatment with 1, 10, and 100 μmol/L ripasudil (*p* = 0.0027, *p* = 0.0065, *p* = 0.0038, respectively). IL-6 and IL-10 decreased after treatment with 100 μmol/L ripasudil (*p* = 0.0080, *p* = 0.0091, respectively). IL-12 decreased after treatment with 10 and 100 μmol/L ripasudil (*p* = 0.0122, *p* = 0.0003, respectively). The levels of CXCL-9 and CXCL-10 did not statistically change after ripasudil treatment (*: *p* < 0.1; **: *p* < 0.01; ***: *p* < 0.001).

To evaluate whether ripasudil alleviates the inflammation caused by HTLV-1 infection in hTM cells, we used a cytometric bead array to detect the levels of inflammatory cytokines and chemokines in the supernatant. After treatment with 100 μmol/L of ripasudil, there was a significant reduction in the level of interleukin (IL)-6, IL-8, IL-10, and IL-12 compared with those in hTM cells without treatment. After the treatment of 10 μmol/L of ripasudil, the level of IL-8 and IL-12 was significantly reduced, and after the treatment of 1 μmol/L of ripasudil, the level of IL-8 was significantly reduced. However, we did not observe any changes in the CXCL-9 or CXCL-10 levels after ripasudil treatment in any of the three groups. TNF-β and CCL-2 levels were not detected in either the ripasudil-treated or untreated groups. Detailed data are shown in Figure 4.

### 2.6. Effect of Ripasudil on NF-κB Activation

**Figure 5:**
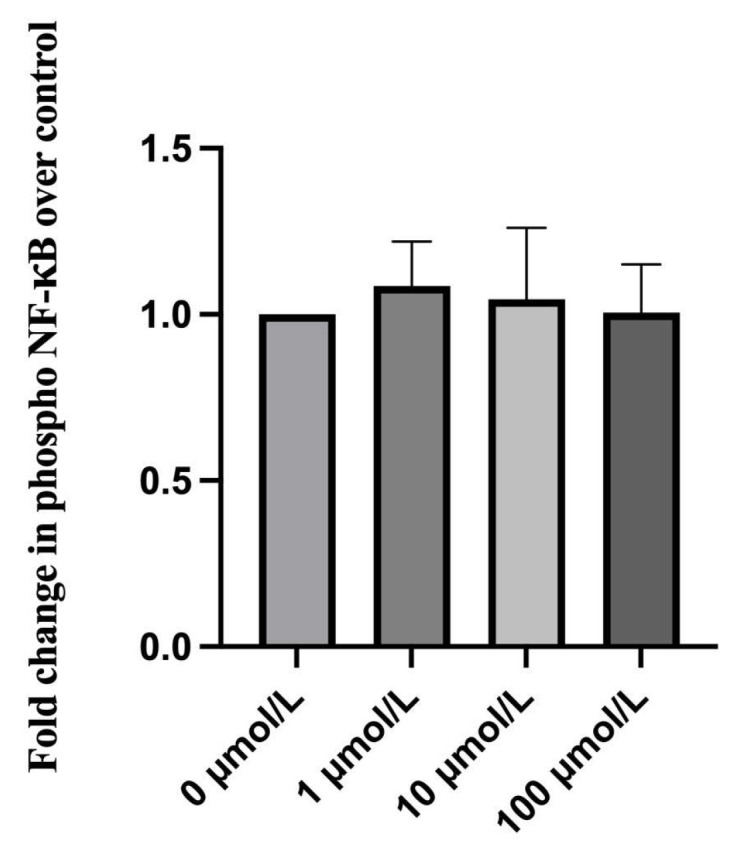
Effect of ripasudil on activation of NF-κB. An enzyme-linked immunosorbent assay was performed to detect the activation of phosphorylated NF-κB after three times transfers. After treatment with different concentrations of ripasudil, phosphorylated NF-κB activation showed no significant change.

Considering the close relationship between NF-κB and inflammatory cytokines and chemokines and the important role of NF-κB in the mechanism of HTLV-1 infection, an enzyme-linked immunosorbent assay (ELISA) was performed to detect the level of phosphorylated NF-κB activation. Compared with hTM cells without any treatment, treatment with ripasudil did not change the level of phosphorylated NF-κB (Figure 5).

## 3. Discussion

Currently, research on secondary glaucoma in patients with HU is limited. As a refractory glaucoma, there are no guidelines or consensuses regarding the treatment of secondary glaucoma in patients with HU. We are trying to find a well-studied and widely used glaucoma medicine for the treatment of secondary glaucoma in HU. Ripasudil, at a concentration of 0.4% (equivalent to 10 μmol/L in our studies), has been approved in Japan since 2014 for the treatment of glaucoma and ocular hypertension [27]. It shows a significant IOP-lowering effect in animals (including rabbits and monkeys) and clinical studies.

Our results indicate that ripasudil alleviated HTLV-1-induced changes in hTM cells, including changes in morphology and function. First, we observed changes in hTM cell shape and F-actin and fibronectin expression (Figure 2 and Figure 3). Inhibiting the ROCK pathway could effectively reduce the phosphorylation of the myosin light chain and actin cytoskeletal organization, resulting in a decrease in actin stress fibers [28]. Previous studies have shown that in primary open-angle glaucoma [28] and glucocorticoid-induced glaucoma [29], ripasudil increases aqueous humor outflow by causing the contraction of the cytoskeleton of TM and Schlemm’s canal cells [30,31]. In our study, ripasudil-induced contraction of cell shape was observed. The distribution of F-actin and fibronectin in infected hTM cells was reduced after ripasudil treatment. These changes may reduce the resistance of aqueous humor outflow, showing promise for the potential use of ripasudil as a lowering IOP treatment for secondary glaucoma of HU.

We examined the effect of ripasudil on hTM cell function. We first examined the effect of ripasudil on cell viability (Figure 1B). Treatment with 1 μmol/L ripasudil increased cell viability. However, the three different concentration treatment groups were not statistically significant compared with the control group. Previous studies have shown the protective effects of ripasudil on cell viability. Chen et al. reported that a ROCK inhibitor promoted the viability of TM cells [32], and other studies have also shown similar results [33,34]. According to our previous study, HTLV-1 infection significantly increased the number of hTM cells [11]. This increase in cell viability may explain why we did not observe a significant protective effect on HTLV-1-infected hTM cells.

Recent studies have focused on the anti-inflammatory effects of ripasudil [25,35]. Therefore, we used a cytometric bead array (CBA) to measure the levels of inflammatory cytokines and chemokines secreted by hTM cells (Figure 4). In our previous study, after being infected by HTLV-1, the levels of inflammatory cytokines and chemokines, including IL-6, IL-8, CCL5, CCL2, CXCL-9, and CXCL-10, significantly increased after HTLV-1 infection [11]. Studies have shown that inflammation of TM tissue plays a crucial role in the increase in IOP in herpetic uveitis [10]. Our previous study suggests that TM cell inflammation caused by HTLV-1 infection may be a key mechanism behind HTLV-1-associated secondary glaucoma in HU. Our current study showed that this increasing trend was attenuated by ripasudil treatment, demonstrating the potential of ripasudil for the treatment of glaucoma secondary to HTLV-1. This finding is consistent with those of several previous studies. They revealed that pretreatment with ripasudil effectively prevented LPS-induced RPE cell inflammation by improving the levels of miR-136-5p and targeting the ROCK2/eNOS signaling pathway [24,36].

Among all the cytokines and chemokines detected, IL-8 was the most sensitive cytokine to ripasudil treatment in our study. IL-8 significantly decreased in all three treatment groups compared with that in the control group. Several studies have shown that IL-8 is significantly increased in patients with primary open-angle glaucoma (POAG) [37], and a correlation between IL-8 and the detection and management of glaucoma has been supported [38]. Moreover, IL-8 recruits macrophages and exhibits profibrotic properties. Recently, Yang et al. showed that IL-8 is associated with fibrotic pathology in patients with idiopathic pulmonary fibrosis [39]. IL-6 is also reduced with the treatment of 100 μmol/L ripasudil in our study. IL-6 is considered an important cytokine associated with the diagnosis and severity of POAG [40,41]. Therefore, reducing the secreted IL-8 and IL-6 levels can at least partly explain the benefit of ripasudil in the treatment of HTLV-1-infected hTM cells, further supporting the feasibility of using ripasudil for the relief of secondary glaucoma in HU. IL-10 is considered as the most important anti-inflammatory cytokine. The observed decrease in IL-10 levels following 100 μmol/L ripasudil treatment might seem to conflict with ripasudil’s anti-inflammatory function. However, IL-10 also plays a critical role in the pathogenesis of POAG. Previous studies have shown that IL-10 genetic polymorphisms are associated with an increased risk of POAG in the Chinese Han population [42], and Mecira et al. have also reported elevated levels of IL-10 in the trabecular meshwork (TM) of glaucomatous eyes [43]. Therefore, the reduction of IL-10 observed in our study should not be interpreted as an indication that the anti-inflammatory action of ripasudil is ineffective. Instead, it further suggests that ripasudil may have a protective effect against the damage caused by HTLV-1 secondary glaucoma. CXCL10 is responsible for attracting immune cells and causing dysfunction of TM by proinflammatory cytokines, which further impede the outflow of aqueous humor [44,45]. In the present study, we observed a decrease in the CXCL9 and CXCL10 levels; however, this was not statistically significant.

Considering the close association between inflammation and NF-κB and the important role NF-κB played in the mechanism of HTLV-1 infection, we then detected phosphorylated NF-κB expression by ELISA (Figure 5). NF-κB activation is required in HTLV-1 infection and ocular hypertension. NF-kB is also important for the fibrosis of human TM [46,47]. According to our previous studies, HTLV-1 infection induced phosphor-p65 NF-κB expression and ultimately improved the inflammation of hTM cells [11]. Several studies have reported the effect of the ROCK pathway on NF-κB. Zhao et al. found that ROCK1/ROCK2 mediated the activation of NF-κB in ethanol-induced impairment of IEB function [48]. Other studies also suggested ROCK-activated NF-κB p65 and a ROCK inhibitor, fasudil, suppressed RhoA/ROCK activation and NF-κB nuclear translocation [49,50]. However, we did not observe any change in phosphorylated NF-κB activation after ripasudil treatment. This can be attributed to the fact that ROCK inhibitor affects NF-κB nuclear translocation instead of the overall level of phosphorylated NF-κB. Nevertheless, this perspective warrants further investigation.

Similar to other types of uveitic glaucoma, the primary treatment goals for secondary glaucoma in HU should focus on controlling inflammation and reducing IOP. From these perspectives, our current study suggests that ripasudil, as demonstrated in vitro, may be a promising treatment. Other clinical studies have found ripasudil effective in glaucoma patients with ocular inflammation [25].

This study has some limitations that should be addressed. While we demonstrated the effects of ripasudil on the morphology and function of HTLV-1-infected hTM cells, this study was confined to in vitro observations, which may not fully represent the complexities of in vivo scenarios. Variables such as cell passage number, cell condition, and timing of treatment could influence the outcomes of our study. Consequently, our findings suggest that ripasudil has potential as a treatment for HTLV-1-associated secondary glaucoma. However, confirming its effectiveness for secondary glaucoma in HU requires extensive animal experimentation and clinical trials. We anticipate that more comprehensive studies will be conducted in the future. Furthermore, it is necessary to consider the complex clinical situation of patients with HTLV-1-associated secondary glaucoma. These patients are often treated with corticosteroids, which can cause changes in immune function and the intraocular environment, potentially affecting the applicability of our findings. Furthermore, the underlying mechanisms of HTLV-1-associated secondary glaucoma remain incompletely understood. Recent studies have highlighted that an imbalance in the Th1/Th17 axis is linked to HTLV-associated myelopathy/tropical spastic paraparesis (HAM/TSP) [51,52], suggesting that HTLV-1-associated diseases may also be considered inflammatory disorders to some extent. Despite this, research focusing on the Th1/Th17 axis in HTLV-1 uveitis (HU) is scarce, and our study did not address this crucial aspect. We anticipate future studies will explore this area and provide deeper insights into the pathogenesis of HU and secondary glaucoma, potentially leading to the identification of novel therapeutic targets.

To conclude, our study showed that ripasudil, a ROCK inhibitor, ameliorated morphological changes by reducing the distribution of F-actin and fibronectin. Ripasudil also reduced inflammation cytokines and chemokines secreted by infected hTM cells, suggesting this glaucoma treatment also has an anti-inflammatory function. Our finding provided a new potential insight for the treatment of HTLV-1 secondary glaucoma in HU.

## 4. Materials and Methods

### 4.1. Cell Culture, In Vitro HTLV-1 Infection, and Treatment

The hTM cells were purchased from ScienCell Research Laboratories (San Diego, CA, USA) and verified by testing the responsiveness of myocilin expression to dexamethasone treatment [53]. HTMCs were isolated from the juxtacanalicular and corneoscleral regions of the human eye. All hTM cells were used within passage 10. The hTM cells were grown in Trabecular Meshwork Cell Medium (ScienCell Research Laboratories, Carlsbad, CA, USA) supplemented with 2% fetal bovine serum (FBS; GE Healthcare Japan, Tokyo, Japan), 1% fibroblast growth supplement, and 1% penicillin–streptomycin. MT-2 cells were used as HTLV-1-infected T cells. MT-2 cells were irradiated with 9000 rads in all experiments, except for the cytometric bead assay. MT-2 was grown in RPMI-1640 (Wako Pure Chemical Corp., Osaka, Japan) supplemented with 10% FBS and 1% penicillin–streptomycin. To trigger HTLV-1 infection, hTM cells were cocultured with irradiated MT-2 for 48 h. Then, MT-2 was removed, and the attached hTM cells were washed, trypsinized, and plated on new culture dishes every 2 days for 96 h. All cells were cultured at 37 °C in a humidified atmosphere containing 5% CO_2_. The hTM cells were treated with different concentrations of ripasudil for 1 h before detection. Phosphate-buffered saline (PBS) was used as a control. Ripasudil hydrochloride was purchased from the Tokyo New Drug Research Laboratories (223645-67-8; Kowa Co., Ltd., Tokyo, Japan).

### 4.2. Measurement of HTLV-1 Proviral Loads

According to the manufacturer’s instructions, an EZ1 Virus Mini kit v2.0 (Qiagen, Hilden, Germany) was used to prepare DNA from each sample. HTLV-1 PVL in HTMCs was measured using quantitative real-time PCR as described previously [54,55,56]. PVL was quantified using the HTLV-1 Tax primer (forward, 5′-CCCACTTCCCAGGGTTTGGA-3′; reverse, 5′-GGCCAGTAGGGCGTGA-3′) and probe (5′-FAM-CCAGTCTACGTGTTTGGA GACTGTGTACATAMRA-3′). Glyceraldehyde-3-phosphate dehydrogenase was used as an internal control.

### 4.3. Cell Viability Assay

Cell viability was assessed using the WST-8 assay with a Cell Counting Kit-8 (CCK-8) (ab228554, Abcam, Burlingame, CA, USA) according to the manufacturer’s protocol. Briefly, hTM cells were cultured overnight in 96-well plates at a density of 5 × 10^3^ cells/well. After ripasudil treatment, CCK-8 was added to the culture medium and incubated for 1.5 h. Absorbance was measured at 450 nm using a microplate reader.

### 4.4. Cell Morphology

The hTM cells were placed on 6-well plates at a density of 1 × 10^4^ cells/well and incubated overnight. Cells were observed using phase-contrast microscopy at 20× magnification and photographed 1 h after the addition of ripasudil. Three images were captured from each well.

### 4.5. Immunofluorescence Assay

The hTM cells were cultured overnight in 24-well plates at a density of 1 × 10^4^ cells/well. After treatment, the culture medium was removed, and the cells were fixed with 4% paraformaldehyde in PBS for 10 min and then washed with 0.1% PBST three times. The cells were permeabilized with 0.3% Triton X-100 in 0.1% PBST for 10 min and blocked with 5% BSA at room temperature for 1 h. Primary antibody F-actin (1:100, ab205, Abcam, Burlingame, CA, USA) and fibronectin (1:200, ab2431, Abcam, Burlingame, CA, USA) were stained at 4 °C overnight. On the second day, the cells were washed three times with 0.1% PBST and then exposed to goat anti-mouse IgG AlexaFluor488 (1:500, 1661231, Invitrogen, Carlsbad, CA, USA) or goat anti-rabbit IgG AlexaFluor488 (1:500, ab150085, Abcam, Burlingame, CA, USA) secondary antibodies at room temperature for 1 h. After washing with 0.1% PBST three times, the cells were mounted with a commercial mounting medium (SlowFade with DAPI, Invitrogen, Carlsbad, CA, USA), and immunofluorescence images were acquired using a BZ-X700 fluorescence microscope (Keyence, Osaka, Japan). Fluorescence intensity measurements were carried out using ImageJ software (version 1.54). Three images from each group were selected for analysis. To ensure unbiased selection based on F-actin and fibronectin staining, images were chosen using DAPI staining to ascertain the number of cells included in the analysis. All images were converted to 8-bit format to facilitate channel separation needed for measurement. The mean gray value was utilized to represent fluorescence intensity.

### 4.6. Western Blot

Cells were seeded on a 6-well plate until the cells formed a confluent monolayer. Proteins were lysed and collected in RIPA (Lot.89900, Thermo Scientific, Carlsbad, CA, USA) with a proteinase inhibitor cocktail (Lot.87786, Thermo Scientific, Carlsbad, CA, USA). The homogenate was centrifuged at 12,000 rpm at 4 °C for 20 min, and the supernatant was stored at −80 °C until needed. Protein concentration was determined with the BCA (Lot. 23227, Thermo Scientific, Carlsbad, CA, USA). Then, 5–20 μg protein was loaded in each lane. Then, the protein was separated on a 4–15% SDS-PAGE gel (Lot. 4561084, Bio-Rad, Hercules, CA, USA) and transferred to the PVDF membranes. After blocking with bullet blocking one buffer (Lot. 13779-01, Nacalai Tesque, Kyoto, Japan) for 5 min at RT, the membrane was incubated overnight at 4 °C with the following primary antibodies: mouse monoclonal to F-actin (1:250, ab205, Abcam, Burlingame, CA, USA), rabbit polyclonal to fibronectin (1:2000, ab2413, Abcam, Burlingame, CA, USA), and mouse monoclonal to GAPDH (1:2000, 171-3, MBL, Nagoya, Japan). The blot was washed with TBST six times and then incubated with either an anti-mouse IgG peroxidase secondary antibody (1:5000, Lot. 9793559, GE Healthcare, Little Chalfont, UK) or an anti-rabbit IgG peroxidase secondary antibody (1:5000, Lot. 9541195, GE Healthcare, Little Chalfont, UK). The blot was developed with ECL solution (Lot. 1705060, Bio-Rad, Hercules, CA, USA) and imaged on ChemiDoc MP. The band intensities were analyzed with ImageJ.

### 4.7. CBA

Inflammatory cytokines and chemokines were measured using a CBA Human Inflammatory Cytokine kit (Lot. 551811, BD Biosciences, San Diego, CA, USA) and a chemokine kit according to the manufacturer’s protocols (Lot. 552990, BD Biosciences). Briefly, hTM cells were seeded in 6-well plates at a density of 1.5 × 10^5^ cells/well overnight. The next day, hTM cells were cocultured with three times MT-2 for 48 h, then MT-2 cells were removed, and the attached hTM cells were washed, trypsinized, and plated on new culture dishes every 2 days for 96 h [11,26]. After cocultivation, the culture supernatants were centrifuged, collected, and then stored at −80 °C until assayed. Then, 50 μL of supernatant was stained with a mixture of human inflammation cytokine and human chemokine capture bead suspension and the PE detection reagent. After incubation for 3 h, the samples were washed and analyzed using BD Analysis Software (BD Biosciences, San Diego, CA, USA). The human inflammatory cytokine and chemokine standards provided in the kit were diluted separately and used to prepare standard curves. The cytokines measured included IL-12p70, TNF-α, IL-10, IL-6, and IL-8, and the chemokines included CCL2, CXCL8 (IL-8), CXCL9, and CXCL10. The sensitivity of cytokines and chemokines detected by the CBA kit was as follows: IL-1b, 7.2 pg/mL; IL-12p70, 1.9 pg/mL; IL-6, 2.5 pg/mL; IL-8, 0.2 pg/mL; IL-10, 3.3 pg/mL; TNF, 3.7 pg/mL; CXCL9, 2.5 pg/mL; CXCL10, 2.8 pg/mL; and CCL2, 2.7 pg/mL.

### 4.8. NF-κB Activity

The level of NF-κB phosphorylation in infected hTM cells was measured using an InstantOne ELISA kit (Cat. No. 85–86083–11, eBioscience, San Diego, CA, USA) according to the manufacturer’s protocol. Absorbance was measured at 450 nm using a microplate reader.

### 4.9. Statistical Analysis

All data are presented as the mean ± deviation. All statistical analyses were performed using Prism 9 (Version 9.4.0). All the experiments in this paper were repeated three times. All comparisons were performed using a one-way ANOVA, except for PVL. Differences in PVL levels were measured using the unpaired Student’s *t*-test. A *p*-value of <0.05 was considered statistically significant.

## Data Availability

All data related to this study are presented and published here.
